# Interoceptive sensibility and alcohol craving in Polish prisoners: the role of alexithymia and emotional dysregulation

**DOI:** 10.3389/fpsyg.2024.1356024

**Published:** 2024-10-07

**Authors:** Dawid Konrad Ścigała, Matteo Angelo Fabris, Elżbieta Zdankiewicz-Ścigała, Krystian Kuc, Claudio Longobardi

**Affiliations:** ^1^Institute of Psychology, The Maria Grzegorzewska University, Warsaw, Poland; ^2^Department of Psychology, University of Turin, Turin, Italy; ^3^Faculty of Psychology, SWPS University, Warsaw, Poland

**Keywords:** alcohol dependence, alcohol craving, inmates, alexithymia, emotion regulation, interoceptive sensibility

## Abstract

**Introduction:**

Alcohol craving, characterized by a strong desire or compulsion to consume alcohol, is a prominent symptom of substance dependence syndrome. Research indicates that alcohol craving is a significant factor leading to the termination of abstinence. The mechanisms underlying the activation of alcohol craving remain not fully understood. The urge to reach for alcohol may be stimulated by emotions, memories, thoughts, or bodily sensations, as well as external factors. It has been postulated that individuals with high levels of interoceptive sensibility tend to exhibit a high degree of alexithymia and emotion dysregulation in the context of alcohol craving. Deficits in identifying and verbalizing emotions, along with an operational thinking style, facilitate alcohol consumption by impeding accurate insight into one’s mental state, thereby hindering the comprehension of bodily states, emotions, and the regulation of self.

**Method:**

This study involved 160 inmates incarcerated in a prison in Poland, awaiting participation in therapy for individuals with substance dependence following psychiatric diagnosis. Four questionnaires were used in the study: multidimensional Assessment of Interoceptive Sensibility (MAIA) for interoceptive sensibility, Toronto Alexithymia Scale (TAS-20) for alexithymia, Difficulties in Emotion Regulation Scale (DERS) for emotional dysregulation, and the Penn Alcohol Craving Scale (PACS) for alcohol craving assessment.

**Results:**

The results of the study are as follows: the study findings indicated that alexithymia and emotional dysregulation significantly mediates the relationship between interoceptive sensibility and alcohol craving. The indirect effect for both factors was found to be significant, similar to the indirect effect observed for alexithymia as an mediator. However, in the case of emotional dysregulation, no significant indirect effect was observed.

**Conclusion:**

Our study provides insights into the potential contribution of interoceptive sensibility to the heightened risk of alcohol dependence. Specifically, impaired interoceptive sensibility may be associated with the development of alexithymia and emotional dysregulation, potentially rendering individuals more susceptible to alcohol craving. Interoceptive sensibility could serve as a prerequisite for the cultivation of positive emotional processing skills.

## Introduction

Alcohol consumption remains a global emergency and can cause significant harm to people’s health, especially when consumption is excessive or addictive (Puddephatt et al., 2022). Data from the World Health Organization (2019) indicates that alcohol is the cause of around 3 million deaths, accounting for around 5% of all deaths worldwide. Prisoners are a population group that is particularly at risk of reporting problems with alcohol consumption or dependence (Baranyi et al., 2022; Pape et al., 2021). In Poland, for example, it is estimated that around 7 percent of prisoners report a diagnosis of alcohol dependence (Stawińska-Witoszyńska et al., 2019). The prison context is important for identifying cases of problematic alcohol use that may escape medical treatment outside prison. In addition, the prison context provides a treatment option that may not only improve the subject’s psychological adjustment to prison, but may also prevent further relapse or criminal recidivism once the prison experience is over (Schramm-Sapyta et al., 2023). However, in order to promote appropriate assessment and relevant psychological and/or pharmacological treatment, it is important to identify risk factors associated with increased symptoms of problematic alcohol use in prison. An analysis of available study results leads to the conclusion that the experience of alcohol craving is a common phenomenon among addicted patients, regardless of status and location. During and after treatment, it is experienced by 54–73% of patients (Chodkiewicz et al., 2015). The occurrence of craving is significantly correlated with relapse (Addolorato et al., 2005). Strong craving is associated with impaired control of drinking and includes obsessive thoughts and compulsive alcohol-related behaviors. Importantly, it is also the only symptom of addiction that can occur even after long periods of abstinence (Chodkiewicz, 2014). Given the role of alcohol craving in the etiology of addiction and in the course of treatment or abstinence maintenance, studies that shed light on understanding the specifics of the underlying mechanisms are important (Martinotti et al., 2013). Our study aims to extend current knowledge by investigating the possible relationship between interoceptive sensitivity and alcohol craving, as well as the possible influence of alexithymia and emotional dysregulation.

### Interoceptive awareness and alcohol craving and dependence

Interoception has been defined as the process by which the nervous system perceives, interprets and integrates signals from within the body, creating a moment-to-moment mapping of the body’s internal landscape at *conscious* and *unconscious* levels (Mehling et al., 2018). Interoception is thus defined as a metacognitive ability that encompasses the different dimensions of interoception that can be consciously perceived and accessible to self-evaluation, allowing us to perceive the physiological state of the body, including emotional states (Mehling et al., 2018). Recent reviews (Khalsa et al., 2017; Suksasilp and Garfinkel, 2022; Desmedt et al., 2023a,b) highlighted the complex, multifaceted nature of interoception, the diverse neural systems and processing levels that are involved in it, and the importance of a multidimensional approach to measurement. Garfinkel et al. (2015) proposed a three-dimensional model of interoception, which includes interoceptive accuracy (the ability to detect and track internal sensations accurately), interoceptive sensibility (self-reported tendency to focus on internal sensations and the detection ability [the type of measurement used in the present study]), and interoceptive awareness (the correspondence between objective interoceptive accuracy and self-report interoceptive sensibility). Given such complexity, it is not surprising that there is conflicting scientific evidence suggesting interoceptive deficits associated with alexithymia and alcohol abuse or alcohol craving.

Based on current research, interoception is linked to the experience of emotions, the processing of emotional stimuli, and the activation of brain structures responsible for monitoring signals indicating physiological and emotional states (Herbert et al., 2011). There is growing empirical support suggesting that interoception forms the foundation for motivation, emotion, social cognition, and human self-awareness (Tsakiris and Critchley, 2016). Several studies seem to indicate a link between interoceptive awareness and alcohol dependence (Price et al., 2019; Wiśniewski et al., 2021, 2023). In particular, a deficit in interoceptive abilities could lead to an increase in alcohol abuse (for a detailed review, see Wiśniewski et al., 2021). The relationship between interoception and substance abuse appears to be complex and bidirectional (Wiśniewski et al., 2021). In general, the literature attributes an important role to interoception in homostatic processing. The allostatic dysregulation induced by repeated alcohol consumption could plausibly be maintained by altered interoceptive regulatory mechanisms that introduce the disparity between the anticipated and actual interoceptive state. However, despite these premises, research on interoceptive sensibility in people with alcohol dependence is still underdeveloped, and there is little research on possible mediating factors. In this study, we will attempt to expand our knowledge by testing a possible mediating role of alexithymia and emotional dysregulation, two constructs strongly associated with interoceptive sensibility and possibly related to forms of dependence.

### Interoception, alexithymia and alcohol craving

Interoception is closely linked to emotional processing (Schuette et al., 2021). Emotions are the result of physiological reactions in response to stimuli from the environment. Therefore, the ability to cognitively name interoceptive signals is important for the development of emotional experiences (Schuette et al., 2021). Furthermore, the neuroscience literature has shown that interoceptive and emotional processing occur in overlapping brain structures (namely the insula and the anterior cingulate cortex) (Ernst et al., 2014). Given the interdependence of physiological and emotional states, it is therefore important for shaping of emotional experiences that individuals are able to recognize and interpret their body signals. Since emotional states are thought to arise from physiological changes in the body, it follows that our ability to perceive these changes based on interoceptive signals allows for more accurate labeling of our emotions. Given the role of interoceptive sensibility in the conscious perception of our internal states and the identification and regulation of physiological and affective arousal, it is possible to understand how deficits in these abilities negatively impact emotional functioning (Murphy et al., 2018). A number of studies indicate that one of the neuronal correlates of alexithymia is reduced neuronal activity in the emotional attentional system, which includes the amygdala, sphenoid bend and occipital cortex. Since the amygdala is involved in eliciting emotional responses and initiating AUN changes (Barrett et al., 2007), reduced amygdala activity in response to emotional stimuli will lead to a reduced physiological response and impaired somatosensory remapping (remapping). As a result, individuals who have difficulty identifying feelings may also have less access to bodily information on which to base spontaneous emotional responses (Donges and Suslow, 2017). Kano et al. (2007) showed that people with high levels of alexithymia exhibit higher levels of anxiety and higher levels of adrenaline during visceral stimulation than people without alexithymia. The authors hypothesized that individuals exhibiting high levels of alexithymia are more sensitive to unpleasant bodily sensations, and their higher autonomic reactivity is manifested in increased right insula activity. Individuals characterized by high alexithymia have higher interoceptive sensitivity and elevated AUN activation, which in turn does not correlate with interoceptive accuracy. Dubey and Pandey (2013) showed that alexithymia is associated with both somatic health and mental health problems. One mechanism that may be responsible for this is the asymmetry in experiencing negative emotions over positive ones, characteristic of people with high levels of alexithymia. It is worth mentioning that individual differences with regard to negative–positive asymmetry are stable over time and transitive (Dubey and Pandey, 2013). Consequently, this entails problems with emotion regulation and self-regulation. Physical symptoms of alexithymia could well be associated with somatosensory amplification (Nakao et al., 2002). Those who suffer from alexithymia may either fail to gain sensibility of otherwise typical physiological arousal or demonstrate atypical arousal with consequences for the subjective experience of emotions (Silani et al., 2008). Alexithymia is considered a personality traits characterized by difficulties in identifying individual’s own feelings, difficulties in describing feelings, and an externally oriented thinking style (Preece et al., 2017, 2024). Alexithymia is also considered as a marker for atypical interoception (Murphy et al., 2018) and several studies suggest a negative association between self-report measures of interoceptive sensibility and alexithymia (Trevisan et al., 2019). Overall, these data seem to indicate that individuals with high levels of alexithymia do not have difficulty perceiving their own internal bodily sensations, but rather have greater difficulty interpreting these sensations (Scarpazza et al., 2022). It is possible that situations that trigger emotions are only perceived on a physical level and remain without any emotional implication (Scarpazza et al., 2015). Alexithymia has been found to be associated with various forms of mental disorders in both children (Prino et al., 2019) and adults (Ścigała et al., 2021, 2022), making it a transdiagnostic factor for mental disorders at any age. In particular, research has found a link between alexithymia and various addictive behaviors (Orsolini, 2020), including alcohol dependence (Lyvers and Thorberg, 2024; Kun et al., 2023). Some research suggests that up to 67% of individuals with alcohol dependence have high levels of alexithymia (Thorberg et al., 2019), and several studies find a positive association between alexithymia and craving in relation to alcohol use (Orsolini, 2020; Thorberg et al., 2019, 2020). Overall, researchers and theorists seem to agree that individuals with high level of alexithymia are likely to have difficulty coping with negative moods through self-regulatory strategies such as problem-focused coping, reappraisal, attention shifting or activation of positive emotions due to their difficulties in identifying and describing their emotions (Wiśniewski et al., 2021), and that they may learn to resort to alcohol to cope with these negative emotions and thus reduce perceived psychological distress. From a neuroscience perspective, activation of the insula (which plays a key role in processing signals from the body, including those associated with drug use and addictive behaviors) is related to the search for somatic and emotional relief previously experienced through alcohol consumption. In other words, interoceptive sensibility contributes to the desire to consume alcohol in order to replicate feelings of emotional and physical relief. In this sense, alcohol acts as a temporary psychological regulator to avoid painful emotional states (Wiśniewski et al., 2021).

Furthermore, if there is a significant discrepancy between the accuracy of interoceptive sensibility and sensitivity to such signals, symptoms of high activation, such as anxiety, may occur. Studies suggest that a greater discrepancy between the accuracy of interoceptive sensibility and its sensitivity is associated with symptoms of high activation, and alexithymia may exacerbate this discrepancy (Zamariola et al., 2018). In a recent study, alexithymia was found to be a possible mediating factor between interoceptive sensibility and anxiety in subjects with alcohol dependence (Wiśniewski et al., 2023), suggesting that alexithymia may be a mediating factor between poor interoceptive skills and psychological distress. This is especially true when we consider populations that are particularly at risk of reporting alcohol dependence, such as incarcerated individuals.

### Interoception, emotion regulation and alcohol craving

Based on the fact that alcohol consumption is seen as a possible strategy for coping with negative emotions, it seems important to examine the possible role of emotion regulation in the relationship between interoceptive awareness and alcohol dependence, also because of the strong associations that emotion regulation has with both constructs. The term emotion regulation refers to the ability to modulate the valence, intensity, or timing of one’s emotional experience and expression in accordance with one’s goals and desires (Gross, 1998). In contrast, emotional dysregulation refers to a diminished ability to understand and accept one’s emotions, as well as difficulty controlling impulsive behaviors and acting in accordance with one’s goals when upset (Gratz and Roemer, 2004). Interoceptive awareness and emotion regulation skills seem to be closely linked. In fact, interoception is not only a basic prerequisite for recognizing emotions, but also seems to be an important factor for emotion regulation (Pinna and Edwards, 2020; Wiśniewski et al., 2021). Adaptive emotion regulation strategies can be understood as the individual’s ability to symbolize and express emotional experiences that manifest on the bodily level (Berking and Znoj, 2008), which is why it is of particular importance to investigate the relationship between interoceptive awareness and emotion regulation. In this direction, there is evidence that interoceptive awareness is a positive prerequisite for effective self-regulation of emotionally driven behavior in healthy individuals (Füstös et al., 2013). Specifically, individuals with high levels of interoceptive awareness tend to engage in reappraisal to downregulate negative affect. This suggests that individuals with high levels of interoceptive awareness do not actively suppress emotional arousal (Füstös et al., 2013). Similarly, a study (Roberts et al., 2022) conducted with opiate-dependent individuals showed that therapeutic interventions aimed at increasing interoceptive awareness tended to result in greater reliance on cognitive reappraisal. Consistent with Gross’s (2002) process model, this cognitive coping strategy aims to regulate emotions before they lead to full-fledged emotional responses and is associated with a reduction in psychological distress. These data appear to be consistent with the neuroscience literature supporting the idea that awareness of bodily sensations is crucial to down-regulate negative emotions through reappraisal (Füstös et al., 2013). Recently, a study (Jakubczyk et al., 2020) showed that alcohol-dependent subjects who exhibited high levels of interoceptive awareness tended to report better emotion regulation than alcohol-dependent subjects who exhibited lower interoceptive awareness.

In the literature, there appears to be a strong association between emotional dysregulation and substance dependence, including alcohol dependence (Fox et al., 2008; Stellern et al., 2023; Weiss et al., 2022). These data are consistent with the self-medication hypothesis (Khantzian, 1997), according to which the use of alcoholic substances may be an attempt by the individual to cope with negative psychological states because they lack appropriate abilities to regulate negative emotional states. As suggested by the resource model of self-control (Inzlicht and Schmeichel, 2012), poor emotion regulation can lead to an individual being more likely to resort to substance use due to a reduced ability to inhibit their behavior in emotionally distressing contexts.

Although several studies have identified emotional dysregulation as a potential risk factor for alcohol dependence. Moreover, it seems important to expand the knowledge on the relationship between interoceptive sensibility, alexithymia and emotional dysregulation and alcohol craving. Alexithymia seem to be strongly integrated and play a key role in the transformation of physiological sensations and internal emotional states into self-consciousness.

### Alexithymia and emotion dysregulation

While it is intuitable how interoceptive sensibility may involve the development of both alexithymic traits and emotional dysregulation, more clarity may be required in explaining the relationship between alexithymia and emotional dysregulation from a temporal perspective. Emotion processing theories imply that identifying and naming emotions accurately is the skill that precedes adaptive regulation (Gratz and Roemer, 2004). In this sense, individual with high level of alexithymia, due to their reduced ability to interpret their emotional experiences, are subjects who tend to exhibit poor emotional regulation. In this direction, some scientific evidence seems to support the fact that alexithymia is a factor that precedes the development of emotional dysregulation, thus constituting a risk factor (Ferraro and Taylor, 2021). This perspective is in line with that postulated by Izard et al. (2002), who indicate that emotion recognition and emotion regulation are two consecutive steps in emotion processing. The Gross extended process model of emotion regulation (Gross, 2002) also identifies the detection and acknowledgement of emotions as the first of three consecutive stages: identification, selection of regulatory strategy, and its implementation. In this sense, alexithymia would represent a dispositional tendency to fail in the identification stage of the extended process model of emotion regulation, leading to emotion dysregulation.

### The purpose of the study

Ultimately, the purpose of this paper is to attempt to expand current knowledge of the possible risk factors associated with a diagnosis of alcohol dependence in a particularly vulnerable population, namely prisoners. In particular, we aim to investigate the relationship between interoceptive sensibility and alcohol craving in this population, identifying the possible mediating role of alexithymia and emotion dysregulation, two factors that are strongly associated with interoceptive deficits and, at the same time, recognized as independent predictors of alcohol craving and dependence. In the context of prison isolation, heightened frustration due to the absence of opportunities to satisfy alcohol cravings may intensify the desire for alcohol. Specifically, we posit that interoceptive sensitivity is positively correlated with alcohol dependence among incarcerated individuals. Additionally, we propose that interoceptive sensibility correlates with elevated levels of alexithymia and emotional dysregulation, which, in turn, are associated with increased susceptibility to alcohol craving and dependence. Moreover, we hypothesize a positive association between alexithymia and emotional dysregulation, further amplifying the risk of alcohol craving. Both alexithymia and emotional dysregulation may hinder accurate interpretation of bodily sensations, thereby increasing the likelihood of alcohol use as a means of regulating arousal or inducing pleasurable affective states. This mechanism pertains to both current and anticipated states experienced by the individual. Alcohol is often misperceived as beneficial for improving physical and mental well-being (Chodkiewicz et al., 2015). In our study, we have chosen to use the total score of the Difficulties in Emotion Regulation Scale (DERS) to provide a comprehensive measure of emotional dysregulation. The DERS total score captures the overall burden of difficulties in various facets of emotion regulation, including nonacceptance of emotional responses, difficulties engaging in goal-directed behavior, impulse control difficulties, lack of emotional awareness, limited access to emotion regulation strategies, and lack of emotional clarity (Gratz and Roemer, 2004). This aggregate measure offers a holistic view of emotion regulation capabilities and allows for broader generalizations about the role of emotional dysregulation in relationships with interoceptive sensibility and alcohol craving. Although the subscales provide detailed insights, focusing on the total score ensures a broad assessment of the overall emotional regulation difficulties, which is pertinent given the integrative nature of the constructs examined in this study.

## Method

### Procedure

The study was conducted in accordance with the guidelines of the SWPS University Committee with written informed consent which was obtained from every single participant. The consent of the Senate Committee on the Ethics of Empirical Research with the Participation of People as Subjects of SWPS University No. 5/2019 was obtained for the study. Every element of the procedure was consistent with the 1964 Declaration of Helsinki, as well as with the standards of research ethics. Before the respondents started filling out the questionnaires, they were informed about the method of filling in the questionnaires and that they could opt out of the questionnaire at any stage. The respondents were also told that participation in the study was voluntary and anonymous.

The study was conducted entirely in prison, and the condition for completing the questionnaires was a diagnosis of alcohol dependence made just before taking the study.

### Participants

Only male inmates incarcerated in a prison in Poland, awaiting participation in therapy for individuals with substance dependence, who met the criteria for alcohol dependence, were included in the study. A total of 160 subjects, with a mean age of *M* = 40.63; SD = 11.51, were examined. Qualified for addiction therapy were exclusively those inmates who met the diagnostic criteria for alcohol addiction. Additionally, they were not dependent on other psychoactive substances and did not exhibit mental disorders that would preclude their participation in addiction therapy. Thus, prisoners were selected for the study based solely on the criterion of alcohol dependence, which also determined their placement in a ward where addiction therapy was conducted. Due to GDPR regulations, the specific crimes for which they were serving their sentences were not verified. Eligibility for the study was determined by individuals authorized to conduct therapeutic work with inmates. It is important to note that being convicted and incarcerated does not inherently categorize the group of incarcerated individuals with addiction issues as a distinct group for the analyzed problem. As previously mentioned, the unavailability of alcohol may increase alcohol craving. Alexithymia, as evidenced in several studies (Bagby et al., 2020), is considered a relatively stable personality trait that does not significantly change under conditions of prison isolation. It is known from the literature that among prisoners, alexithymia is significantly correlated with experiences of childhood trauma, depression, anxiety, or suicide attempts (Chen et al., 2017; Hemming et al., 2020).

### Instruments

*Toronto Alexithymia Scale-20* (TAS-20) [Bagby et al., 1994, 2020; in the Polish adaptation by Ścigała et al. (2020)]. It is a self-report questionnaire and consists of 20 statements. Respondents refer to these statements on a five-point Likert scale from 1 – completely disagree to 5 – completely agree. The scale consists of three subscales: (1) Difficulty in Identifying Feeling (DIF), which consists of 7 statements. It is used to assess the difficulty of identifying feelings and distinguishing them from physiological reactions; (2) Difficulty in Describing Feeling (DDF) consists of 5 statements and allows to assess the difficulty in verbalizing feelings and naming emotions; (3) Externally Oriented Thinking (EOT), which consists of 8 items and allows to assess an externally oriented, operational mindset. The analysis of the obtained results is based on the score obtained on a particular scale and the overall result. A subject may score from 20 to 100 points in total. Alexithymia is treated as a dimension, the higher the score on the scale, the higher its level. Scores below 50 points are referred to as low levels of alexithymia, between 51 and 60 points possible alexithymia, and above 61 points are referred to as high levels of alexithymia (Bagby et al., 1994). The questionnaire is characterized by satisfactory reliability as measured by Cronbach’s alpha measure, *α* = 0.86 and by McDonalds Omega, *ω* = 0.85. The individual scales within the questionnaire also showed satisfactory reliability (DIF: *α* = 0.81 ω = 0.70; DDF: *α* = 0.71 ω = 0.64; EOT: *α* = 0.61 ω = 0.71).

*Multidimensional Assessment of Interoceptive Sensibility* (MAIA) [Mehling et al., 2012; Polish adaptation by Brytek-Matera and Kozieł (2015)]. It is a self-report questionnaire for a multidimensional assessment of interoception. Interoceptive awareness as conceptualized within the MAIA is comparable with the construct of self-reported interoceptive sensibility as proposed by Garfinkel et al. (2015). The person in the study refers to the statements presented, marking a correct digit on the six-point Likert scale from 0 – it does not concern me at all, to 5 – it concerns me very much. High scores on individual subscales indicate a high level of interoceptive sensibility. The questionnaire contains 32 statements, assigned to eight subscales: (1) Noticing – sensibility of comfortable, uncomfortable or neutral bodily sensations (e.g., “When I am tense, I notice where in my body the tension is”): (2) Not Distracting – the tendency to distract oneself in order to cope with discomfort (e.g., “I distract myself from the feeling of discomfort”); (3) Not Worrying – the tendency to feel emotional distress associated with physical discomfort (e.g., “When I feel physical pain, I get anxious”); (4) Attention Regulation – the ability to maintain and direct attention to body sensations (e.g., I can focus my attention on my breath without paying attention to what is happening around me”); (5) Emotional Sensibility – sensibility of the connection between bodily sensations and emotional states (e.g., “I notice how my body changes when I’m angry”); (6) Self-Regulation – the ability to regulate distress by paying attention to bodily sensations (e.g., “When I feel overwhelmed, I can find a quiet place inside myself”); (7) Body Listening – the tendency to actively listen to one’s body in order to gain insight into one’s state (e.g., “I’m listening to the information my body is sending about how to act”); (8) Trusting – experiencing one’s own body in the context of a sense of security (e.g., “I feel at home in my own body”). For the Polish language version of the questionnaire, Cronbach’s *α* and McDonalds *ω* for individual subscales is: for the “noticing” factor – *α* = 0.67; ω = 0.79, for the “non-distraction” factor – *α* = 0.55; ω = 0.60, for the “not worrying” factor *α* = 0.67; *ω* = 0.66, for the “attention regulation” factor – *α* = 0.85; ω = 0.82, for the “Emotional sensibility” factor – *α* = 0.87; ω = 0.80, for the “self-regulation” factor – *α* = 0.82; ω = 0.81, for the “listening to the body” factor – *α* = 0.80; ω = 0.82, for the “trust” factor – *α* = 0.91; ω = 0.81 (Brytek-Matera and Kozieł, 2015). An overall score can be calculated by summing all items. Higher scores indicate higher levels of interoceptive awareness.

*Difficulties in Emotion Regulation Scale* (DERS) (Gratz and Roemer, 2004) is a psychometric tool designed to measure difficulties in regulating emotions. This scale allows to identify areas where a person may encounter difficulties in dealing with emotions. The scale consists of 36 questions that assess different aspects of difficulty in regulating emotions. Participants’ answers to individual questions are evaluated on a 5-point Likert scale, where 1 – “almost never” and 5 – “almost always.” The points are added together for each of the six subscales, allowing to calculate an overall score of difficulty in regulating emotions. A higher score on the scale indicates a lower ability to cope within the measured range. In the current study, we utilize the total score of the DERS to capture the overall degree of emotional dysregulation, as this provides a comprehensive assessment of emotion regulation difficulties. The authors of the questionnaire (Gratz and Roemer, 2004) posited the existence of an overall score. This was also confirmed in the study by Lee et al. (2016).

DERS shows good psychometric indicators, confirmed by studies on its reliability and validity. The internal agreement measured by Cronbach’s alpha and McDonald’s omega for the entire scale is *α* = 0.90; *ω* = 0.87, indicating high internal consistency. This scale also has appropriate validity, which means that it accurately measures what it was intended for, i.e., difficulties in regulating emotions.

*Penn Alcohol Craving Scale* (PACS) [Flannery et al., 2003; in the Polish adaptation by Chodkiewicz et al. (2018)]. The scale consists of five questions. It is a tool to test the craving for alcohol. The scale responds to the demand resulting from the classification of alcohol dependence according to the DSM-5 (APA, 2013). The answers are given on a seven-point Likert scale from 0 to 6. Three questions measure the frequency, intensity and duration of cravings, one measures the ability to resist temptation when drinking is possible, and another estimates the severity of the overall craving for alcohol over the past week. The questions were prepared according to the classification. The calculation of the results is based on adding up of the results obtained in the five test items. For the PACS in the Polish adaptation, the following ranges were obtained: 0–3 – low craving severity, 4–9 average, above 10 – high. In the Polish version of the tool, a score above 10 points indicates a high level of craving. The tool is often used in overseas research on alcohol cravings. It is worth emphasizing once again that the authors of the method indicate such a result as a diagnostic for the risk of breaking abstinence and discontinuation of therapy. The scale has good psychometric properties Cronbach’s alpha and McDonald’s omega for the entire scale is α = 0.93; ω = 0.93, the test–retest correlation coefficients were obtained at the significance level of *p* < 0.01, and the correlations with other craving testing methods were significant at the level of *p* < 0.01.

### Data analysis

The first stage of the data analysis was to verify the distribution of the presented variables, whose skewness distributions were in the range of −1 to +1 standard deviation, which suggests that the distribution of the results of the variables does not deviate from the normal distribution (Tabachnick and Fidell, 2013). The next step was to analyze the relationship between the variables using Pearson’s correlation coefficient as an introduction to further analyses. The analysis of the data focused primarily on the verification of the theoretical model, which was verified using the non-standard macro Process 4.2 model 6 (Hayes, 2018). This solution was chosen because the presented model allows to verify the influence of two mediators, which are strongly correlated with each other, as is the case with alexithymia and emotional dysregulation. In addition, the serial multiple mediation model allows to verify the relevance of each mediator separately, as well as both at the same time, and compares the strength of the effects with each other. In accordance with the approach proposed by Hayes (2012), the statistical significance of the indirect mediating effects of variables upon the bootstrap method is evaluated based on whether the point estimate of the mediating variable is zero within a 95% bias-corrected and accelerated confidence interval (BCa CI). Accordingly, to consider an indirect effect statistically significant, the specified 95% bias-corrected bootstrap confidence interval should have contained “0.” Hayes (2013, 2018) proposed that 10,000 bootstrap samples be utilized for mediation analyses in the test of serial multiple mediation. Consequently, the data derived from 10,000 bootstrap samples were utilized in the present study.

## Results

At the outset, focusing on assumptions regarding the relationship between Interoceptive Sensibility, alexithymia and difficulties in emotion regulation and the level of alcohol cravings, they can be considered fulfilled because there is a moderate positive correlation between alexithymia and difficulties in emotion regulation and alcohol cravings and a negative correlation with interoceptive sensibility (Table 1). In addition, it is worth noting that the level of alexithymia obtained in the group of prisoners is similar to the level defined by the authors of the tool as meeting the alexithymia criterion (Bagby et al., 2020), and also similar to the level obtained in another group of addicts in the study of adaptive questionnaires to measure the level of alexithymia (Ścigała et al., 2020) (Table 1).

**Table 1:** Means, standard deviations and bivariate correlations of variables under study.

	*M*	*SD*	*Skew*	*Kur*	1	2	3	4
1. Alexithymia	58.57	12.28	−0.741	0.429	-			
2. Interoceptive sensibility	22.13	4.99	−0.877	1.191	−0.179*	-		
3. Difficulties in emotion regulation	98.40	18.98	−0.022	0.323	0.416*	−0.261**	-	
4. Alcohol craving	9.50	7.05	0.389	−0.299	0.434**	−0.391**	0.375**	-

M, mean; SD, standard deviation; Skew, skewness; Kur, kurtosis. **p < 0.001; *p < 0.05.

**Figure 1:**
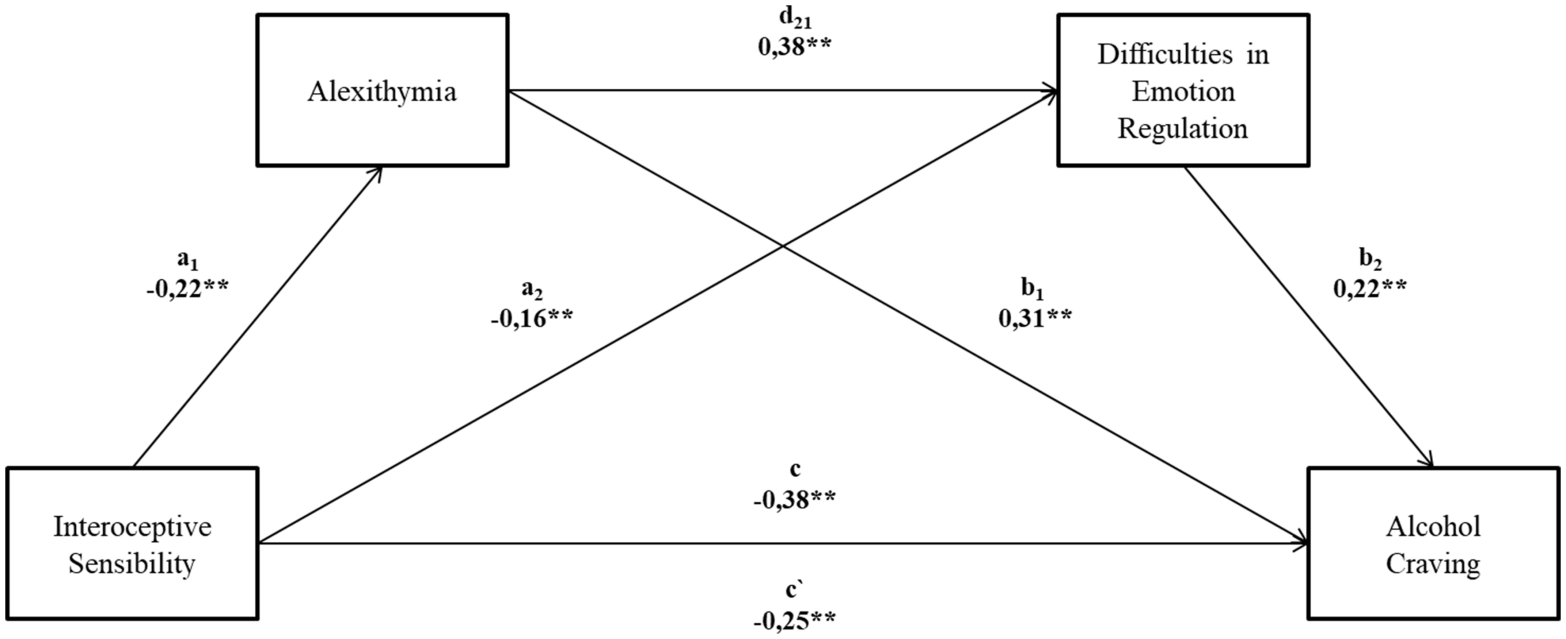
Serial-multiple mediation model for the relation between interoceptive sensibility and alcohol cravings, alexithymia and difficulties in regulating emotions as a mediators. (c) A direct effect of the impact of interoceptive sensibility on the alcohol craving level. (a1, b1) An indirect effect of the impact of interoceptive sensibility on the alcohol craving level, including alexithymia. (a2, b2) An indirect effect of the impact of interoceptive sensibility on the alcohol craving level, including difficulties in emotion regulation. (a1, d21, b2) An indirect effect of the impact of interoceptive sensibility on the alcohol craving level, including alexithymia and difficulties in emotion regulation. (c`) A direct effect of the impact of interoceptive sensibility on the alcohol craving level, taking account of the impact of both mediators. All values on the figure are standardized; **p < 0.001; All non-standardized coefficients and standard errors are included in Table A1.

However, the main purpose of the analysis was to test the relationship between alexithymia and difficulty regulating emotions with the relationship between interoceptive sensibility and alcohol craving (Figure 1). The tested model is well fitted to the data *F*(3.143) = 23.10; *p* < 0.001 and explains the 32.7% variation in alcohol cravings. When analyzing individual relationships, it is worth starting with the direct relationship between interoceptive sensibility and alcohol craving, which is statistically significant (*c* = −0.38; *p* < 0.001). So is the relationship between interoceptive sensibility and alexithymia (a1 = −0.22; *p* < 0.01) and interoceptive sensibility and difficulties in regulating emotions (a2 = −0.16; p < 0.01). The relationship between the two mediators is also significant (d21 = 0.38; p < 0.01). The relationship of both mediators to alcohol cravings is also significant and is, respectively, for alexithymia (b1 = 0.31; *p* < 0.01) and for difficulties in regulating emotions (b2 = 0.22; *p* < 0.01). When mediators are added into the model, the relationship between interoceptive sensibility and alcohol cravings decrease to (c’ = −0.25; *p* < 0.01). The indirect effect analysis is based on the verification of confidence intervals with 10,000 sampling based on the bootstrapping method. All non-standardized coefficients and standard errors are included in Table A1 in Appendix. Focusing on the first indirect effect, i.e., when only alexithymia is a mediator (a1,b1), this effect has been shown to be significant: point estimate *b* = 0.068.95% CI [−0.14; −0.01]. The second indirect effect for difficulties in regulating emotions (a2, b2) was found to be statistically insignificant point estimate *b* = 0.035. 95% CI [−0.09, 0.01]. On the other hand, the effect considering both mediators at the same time is statistically significant (a1, d21, b2) point estimate b = 0.0186. 95% CI [−0.04; −0.01]. The total effect (c – c’) is also statistically significant: point estimate *b* = − 0.56. 95% CI [−0.33; −0.38].

## Discussion

The objective of this study is to examine the theoretical model that assumes a relationship between interoceptive sensibility and alcohol craving in a sample of incarcerated individuals who are at a relatively high risk of alcohol dependence. Furthermore, the study aims to contribute to the existing body of knowledge regarding the potential mediate relation of emotional dysregulation and alexithymia on the relationship between interoceptive sensibility and alcohol craving.

The proposed theoretical model assumed a significant relationship between interoceptive sensibility and alcohol craving, and this relationship is indeed significant. This finding appears to align with recent studies indicating a potential role for interoceptive sensibility in the development of addiction, particularly alcohol dependence (Price et al., 2019; Wiśniewski et al., 2023). However, the existing literature on the relationship between interoceptive sensibility and alcohol craving is not well developed, particularly when considering certain populations that are particularly at risk, such as prisoners. These conclusions, among other things, allowed us to construct the mediation model we presented, which turned out to be statistically significant, also indicating that incarcerated individuals diagnosed with alcohol dependence tend to report greater deficits in their interoceptive skills, particularly in interoceptive sensibility. Given the evidence that interoceptive sensibility plays a significant role in emotional processing (Pollatos and Schandry, 2008), we sought to explore the extent to which alexithymia and emotional dysregulation might act as mediators in the relationship between interoceptive sensibility and alcohol craving.

Our model, which assumed that alexithymia would play a mediating role in the aforementioned relationship, is significant. Specifically, a deficiency in interoceptive abilities is linked with increased alexithymia, which, in turn, is associated with higher alcohol craving symptoms. The extant literature appears to indicate that alexithymia may serve as a marker for atypical interoceptive sensibility (Murphy et al., 2018). Additionally, our theoretical model assumes a potential mediating role of alexithymia in the relationship between interoceptive sensibility and alcohol craving.

Consequently, this finding appears to suggest that incarcerated individuals with alcohol craving tend to exhibit diminished interoceptive competence, which in turn gives rise to elevated alexithymia. Consequently, elevated levels of alexithymia are typically associated with heightened alcohol craving. A number of studies have identified an association between alexithymia and alcohol dependence (Lyvers and Thorberg, 2024; Thorberg et al., 2019; Kun et al., 2023). However, our study contributes to the existing literature by suggesting that alexithymia may act as a mediator in the relationship between alcohol craving and interoceptive sensibility. It can thus be postulated that individuals with poor interoceptive abilities may be less capable of recognizing and identifying their emotions. In this condition, individuals may attempt to avoid painful feelings and emotions that are predominantly experienced at the somatic level by turning to alcohol as a potential psychological regulator (Wiśniewski et al., 2021). In a recent study, Lyvers and Thorberg (2024) examined the relationship between alexithymia, emotion regulation, and interoceptive sensibility in the context of alcohol dependence. The results of the statistical analyses indicated that there was a strong correlation between alcohol dependence and alexithymia, as well as sensitivity to reward. Conversely, there was a negative correlation between alcohol dependence and emotion regulation. Notably, no relationship was observed between alcohol dependence and interoceptive sensibility. It is noteworthy that the aforementioned study examined the association with alcohol dependence. The objective of this study was to analyze the association between interoceptive sensibility and alcohol craving. Alcohol craving is defined as a subjective desire or feeling of compulsion to consume the substance, or a strong need to experience pleasure from its use, or a compulsive desire (Chodkiewicz et al., 2015). The lower the level of interoceptive sensibility, the more likely it is that the misinterpretation of bodily signals will become conscious. Furthermore, our theoretical model assumed emotional dysregulation as a potential mediator in the relationship between interoceptive sensibility and alcohol craving. The neuroscience literature indicates that interoceptive sensibility plays a fundamental role in the ability to down-regulate negative emotions. Our research builds upon this line of inquiry by demonstrating a negative correlation between interoceptive sensibility and emotional dysregulation. Consequently, the ability to recognize one’s bodily sensations is of paramount importance for the activation of appropriate coping strategies. Consequently, emotional dysregulation was found to be associated with an increase in alcohol craving. This finding is consistent with previous literature (Fox et al., 2008; Stellern et al., 2023; Weiss et al., 2022), which indicates that emotional dysregulation is a significant risk factor for the development of alcohol dependence. The self-medication hypothesis posits that individuals with emotional dysregulation may be more likely to turn to alcohol as a coping strategy to regulate their emotions and alleviate negative mental states, thereby increasing the risk of receiving a diagnosis of alcohol dependence.

Finally, our theoretical model suggested a sequential mediation effect, in which alexithymia was positively associated with emotional dysregulation, which in turn was positively associated with alcohol craving symptoms in incarcerated individuals. The results of our study are consistent with recent evidence suggesting that alexithymia is a precursor to emotional dysregulation (Izard et al., 2011; Ferraro and Taylor, 2021). In this context, the capacity to recognize one’s own emotional states appears to be a fundamental prerequisite for the development of effective emotion regulation strategies, thereby reducing the likelihood of emotional dysregulation.

Our model assumes positive correlation between alexithymia and emotional dysregulation which, in turn, mediates the relationship between alexithymia and alcohol craving. Which may imply, that low levels of interoceptive sensibility are associated with increased alcohol craving in incarcerated individuals. This relationship appears to be mediated by alexithymia and emotional dysregulation.

In conclusion, our research suggests that interoceptive sensibility might plays a significant role in the risk of developing alcohol craving or maintaining the syndrome. In particular, interoceptive sensibility may prevent individuals from naming their emotions, thus preventing the recognition and identification of their emotions. This deficit represents the core feature of alexithymia. Consequently, deficiencies in interoceptive abilities may elevate the probability of developing alexithymia, which in turn impedes the formation of effective emotion regulation techniques. Consequently, those with inadequate interoceptive abilities are at a heightened risk of developing alexithymia, which in turn can exacerbate emotional dysregulation. As a result of their inability to distinguish between physical and emotional sensations and to verbalize their emotions, those affected may find it challenging to develop the necessary emotional regulation skills. In this state, the individual may turn to alcohol as a means of escaping negative emotions, thereby establishing alcohol as a kind of “psychological regulator.” In this context, the efficacy of alcohol in reducing the arousal associated with negative emotions that the individual is unable to recognize, name, or regulate becomes a maladaptive coping strategy, thereby reinforcing alcohol dependence.

## Limits and future directions

Despite the contribution of our study to the current literature, the results must be read with serious consideration of the limitations of the study. Although all subjects were formally diagnosed with alcohol dependence, the sample we recruited cannot be considered representative of the prison population. Therefore, future studies could recruit larger clinical samples that are more representative of the prison population and replicate the study in different cultural contexts. Furthermore, in our study we used exclusively self-report questionnaires. The use of self-report could influence the subjects’ responses due to the effect of social desirability, the ability to understand the text and the actual sensitivity of the subjects to their own emotional functioning. Moreover, it is worth noting that although the MAIA questionnaire used in the study has acceptable psychometric properties and the tool’s authors permit the use of the total score, there are some discrepancies in other publications regarding the factorial structure of the instrument. Future studies could therefore use third-party observers or other measurement tools. In addition, our study is cross-sectional, so it is not possible to examine linear causal relationships between the variables. Future studies could therefore use a longitudinal research design to better understand this aspect. Finally, future studies may include in the research protocol additional variables potentially linked to alcohol craving, such as other personality traits or environmental stressors, enriching the model and therefore studying more complex models.

## Practical implications

Our study has some practical implications. First, our theoretical model suggest the importance of considering certain variables related to emotional processing skills when assessing incarcerated individuals suffering from alcohol dependence. Specifically, our model indicate the importance of assessing interoception and relat1ed outcome factors such as alexithymia and emotional dysregulation. These features could become important elements on which forms of psychotherapeutic intervention may be built to improve the psychological state of incarcerated individuals and thus reduce the development of alcohol dependence. In this direction, evidence suggests (Price et al., 2019) that training aimed at developing interoceptive sensibility tends to improve emotional competence and thereby reduce substance addictions. Therefore, working on interoceptive sensibility may help subjects to develop more appropriate emotional skills that, in turn, may reduce the risk of developing or maintaining alcohol dependence.

## Conclusion

To summarize, our study seeks to extend our knowledge of the relationship between interoceptive sensibility and alcohol dependence in a sample of incarcerated individuals by examining the potential mediating role of alexithymia and emotional dysregulation. Ultimately, our theoretical model and results supports the role of interoceptive sensibility in increasing the risk of alcohol dependence. In particular, impaired interoceptive sensibility could contribute to the onset of alexithymia and emotional dysregulation, thereby increasing individuals’ susceptibility to alcohol dependence. Interoceptive sensibility could be a prerequisite for the development of positive emotional processing skills. Poor interoceptive sensibility skills could lead to the subject developing alexithymia and thus emotional dysregulation. In this case, the person would not be able to distinguish emotional states from physical states. They would have difficulties recognizing and naming emotions and thus adaptively regulating them. In this way, the affected person could resort to alcohol as a ‘psychological regulator’ to reduce the activation of negative emotional states, thus increasing the dependence on alcohol to reduce emotional distress.

## Data Availability

The raw data supporting the conclusions of this article will be made available by the authors, without undue reservation.

## Appendix

**Table A1:** Standardized and non-standardized coefficients for serial-multiple mediation model for the relation between interoceptive sensibility and alcohol cravings, alexithymia and difficulties in regulating emotions as a mediators.

Variable	Variable	Standardized coefficients	Non-standardized coefficients	Standard errors	*t*	*p*
Interoceptive Sensibility	Alexithymia	−0.22	−0.5756	0.2111	2.7272	0.0072
Interoceptive Sensibility	Difficulties in Emotion Regulation	−0.16	−0.6296	0.3072	2.0490	0.0423
Alexithymia	Difficulties in Emotion Regulation	0.38	0.5886	0.1179	4.9922	0.0001
Interoceptive Sensibility	Alcohol Craving	−0.26	−0.3781	0.106	3.5653	0.0005
Alexithymia	Alcohol Craving	0.31	0.1757	0.0434	4.0430	0.0001
Difficulties in Emotion Regulation	Alcohol Craving	0.22	0.0815	0.0284	2.8748	0.0047
Interoceptive Sensibility	Alcohol Craving	−0.38	−0.5581	0.1143	4.8818	0.0001

t, *t-*tests; *p*, level of significance.
